# Difference in the prevalence of advanced colon adenoma between patients with gastric neoplasm and healthy people: A STROBE-compliant study

**DOI:** 10.1097/MD.0000000000029308

**Published:** 2022-05-27

**Authors:** Myeongseok Koh, Min-Chan Kim, Jin Seok Jang

**Affiliations:** a Department of Internal Medicine, Dong-A University Hospital, Dong-A University College of Medicine, Busan, South Korea; b Department of Surgery, Dong-A University Hospital, Dong-A University College of Medicine, Busan, South Korea.

**Keywords:** advanced colon adenoma, colon polyp, colonoscopy, gastric neoplasm

## Abstract

We compared the prevalence of adenoma and cancerous colon polyps in patients undergoing endoscopic removal or gastric surgery for gastric adenoma or gastric cancer and in healthy individuals.

The medical records of 707 patients with gastric neoplasm and 798 age- and sex-matched healthy subjects were retrospectively analyzed between January 2010 and July 2018. The clinicopathological characteristics, prevalence of colorectal neoplasm diagnosed by colonoscopy, and risk factors for colorectal polyps were also investigated.

When comparing the two groups, the prevalence of overall colorectal polyps and its distribution was not different between the two groups (54.0% vs.49.5%, *P* = .079), whereas, the number of colon polyps (1.20 ± 1.71 vs 0.99 ± 1.54, *P* = .015) and the maximal size (3.53 ± 6.14 vs 2.08 ± 2.88, *P* < .001) were significantly larger in the gastric neoplasm group. The prevalence of advanced colon adenoma was significantly higher in the gastric neoplasm group (10.7% vs 3.8%, *P* < .001). Risk factors such as elevated glucose levels and the presence of gastric neoplasm were related to the prevalence of all colon polyps. The presence of gastric neoplasm is an important risk factor for advanced colon polyps.

Patients with gastric neoplasms had a significantly higher prevalence of advanced colon adenoma. Advanced colon adenoma is associated with the chain from benign adenomas through malignant altered adenomas to advanced colon cancer. Thus, patients with gastric neoplasm are regarded as a high-risk group for colorectal cancer and are recommended for screening colonoscopy at the time of diagnosis.

## 1 Introduction

Gastric cancer (GC) is the fifth most common cancer and the second most common cause of cancer-related deaths worldwide.^[1]^ However, significant regional differences were observed in its incidence, with East Asia, Eastern Europe, and parts of Latin America having the highest incidence, and South Asia, North Africa, East Africa, Australia, and North America the lowest.^[2,3]^

As early detection is associated with better outcomes, screening programs are conducted in countries with a high prevalence of GC.^[4]^ In Korea, screening was initiated in 1999 and involves upper endoscopy or upper gastrointestinal series for patients aged ≥40 years every 2 years. As a result of this screening program, > 50% of GCs are diagnosed at an early stage in Korea. Therefore, additional synchronous neoplasms should be detected for further treatment and overall patient survival. GC associated with additional synchronous neoplasms has been reported based on its clinicopathological features and patient outcomes.^[5–7]^ Colorectal cancer is one of the most frequent synchronous cancers among patients with primary GC.^[6–9]^ The incidence of colon cancer has been rapidly increasing in recent decades in Asian countries, making it the third most common cause of cancer-related deaths in Korea.^[10]^

In this study, the prevalence of colon polyps and advanced colon adenoma was investigated in patients who underwent endoscopic removal or gastrectomy for gastric adenomas or early gastric cancers (EGC) and colonoscopy for screening and in healthy control participants undergoing simultaneous colonoscopy for a routine checkup.

## 2 Materials and methods

### 2.1 Participants

Clinical records, including pathological findings, of 3067 patients who underwent endoscopic removal or gastric surgery for gastric adenoma or GC between January 2010 and July 2018 at the Dong-A University Hospital were retrospectively reviewed. Among these patients, 707 who underwent colonoscopy were identified (the study group, gastric neoplasm group). In addition, age- and sex-matched healthy participants undergoing concurrent esophagogastroduodenoscopy and colonoscopy for a general checkup during the same period at the Center for Health Promotion of Dong-A University Hospital were also recruited (the control group). Patients were included if the diagnosis of gastric adenoma or GC was confirmed histologically.

Individuals were excluded from both groups if they had any of the following: (1) history of colorectal polypectomy or surgery; (2) family history of colorectal cancer; and (3) warning symptoms of colorectal cancer (abdominal pain, palpable mass, bowel habit changes, hematochezia). Individuals were excluded from the control group if they had any of the following: (1) gastric adenoma or adenocarcinoma in concurrent esophagogastroduodenoscopy; (2) history of gastric polypectomy or surgery; and (3) family history of GC. The number, size, location, and histology of adenomas were investigated for both gastric and colonic adenomas. The distribution of colonic adenomas was classified into the right colon (cecum, ascending colon, hepatic flexure, and transverse colon) and left colon (splenic flexure, descending colon, sigmoid colon, and rectum). The size of each adenoma was estimated by eye measurements using open biopsy forceps. The histology of gastric adenomas was classified as adenoma with high-grade dysplasia and low-grade dysplasia. GC was classified as EGC or advanced gastric cancer (AGC). Advanced colonic adenomas were defined as tubular adenomas with diameters > 1 cm, adenoma with a villous component, adenoma with high-grade dysplasia, and adenocarcinoma. Body mass index (BMI) was calculated by dividing weight (kg) by height squared (m^2^). Total cholesterol, low-density lipoprotein (LDL)-cholesterol, high-density lipoprotein (HDL)-cholesterol, and fasting glucose levels were measured after a fasting period of 12 hours.

### 2.2 Statistical analysis

The frequency of colorectal neoplasms was compared between the gastric neoplasm group and control group. In the two groups, categorical variables were compared using Pearson's chi-squared method and continuous variables by Student t-test. A multivariate logistic regression model was used to evaluate risk factors for colorectal neoplasms. For each variable, odds ratios (ORs) and 95% confidence intervals (CIs) were determined. A *P*-value of < .05 was considered to indicate statistical significance. Database management and statistical analyses were performed using SPSS 21 for Windows (Inc., Chicago, IL).

### 2.3 Ethics statement

The present study protocol was reviewed and approved by the Institutional Review Board of Dong-A University Hospital (DAUHIRB-19–015) on January 25, 2019.

## 3 Results

Table 1 shows the baseline characteristics of the patients in the gastric neoplasm group and control group. A total of 707 patients were enrolled in the study group and 798 healthy participants in the control group. The mean age of patients in the study group was 58.85 ± 9.93 years, and 494 (69.9%) were men. The mean BMI, HDL cholesterol, and CA 19-9 were not significantly different between the two groups. However, total cholesterol and LDL-cholesterol levels were higher in the control group, whereas fasting glucose and carcinoembryonic antigen levels were higher in the study group.

**Table 1:** Baseline characteristics in the study group and the control group.

	Study group (n = 707)	Control group (n = 798)	*P* value
Age (yr, mean ± SD)	58.85 ± 9.93	57.53 ± 6.12	.456
Sex, male:female	494: 213	550: 248	.690
BMI (kg/m2; mean ± SD)	24.22 ± 10.27	24.30 ± 3.18	.835
*Serum level*			
Total cholesterol (mg/dL; mean ± SD)	181.32 ± 87.08	205.13 ± 36.93	**< .001**
Low density lipoprotein (mg/dL; mean ± SD)	115.66 ± 37.26	126.14 ± 34.45	**.003**
High density lipoprotein (mg/dL; mean ± SD)	56.39 ± 25.69	56.39 ± 14.91	1.000
Glucose (mg/dL; mean ± SD)	108.31 ± 32.13	92.71 ± 19.85	**< .001**
CEA (ng/mL; mean ± SD)	3.04 ± 6.94	2.05 ± 1.39	**.001**
CA 19–9 (U/mL; mean ± SD)	12.75 ± 55.98	9.68 ± 11.26	.312
*Colonoscopy*			
Colon polyp n (%)			.079
YES	382 (54.0%)	395 (49.5%)	
NO	325(46.0%)	403 (50.5%)	
Colon polyp, (Number., mean ± SD)	1.20 ± 1.71	0.99 ± 1.54	**.015**
Colon polyp site, n (%)			.361
Right	172 (24.3%)	173 (21.7%)	
Left	102 (14.4%)	108 (13.5%)	
Both	108 (15.3%)	114 (14.3%)	
Maximum size of colon polyp, (mm, mean ± SD)	3.53 ± 6.14	2.08 ± 2.88	**< .001**
Advanced adenoma, n (%)			**< .001**
YES	76 (10.7%)	30 (3.8%)	
NO	631 (89.3%)	768 (96.2%)	
Polyp pathology, n (%)			**< .001**
Tubular adenoma	303 (42.9%)	292 (36.6%)	
Tubulovillous/villous adenoma	19 (2.7%)	10 (1.3%)	
Serrated adenoma	15 (2.1%)	16 (2.0%)	
Adenocarcinoma	14 (2.0%)	1 (0.1%)	

BMI = body mass index, CA 19–9 = carbohydrate antigen 19–9, CEA = carcinoemcryonic antigen, SD = standard deviation.

The study group was further divided into three groups (gastric adenoma, EGC, and AGC) and then analyzed. Between-group differences in age, BMI, and LDL cholesterol levels were significant. However, no differences were found in the colonoscopic findings (Supplementary Digital Content Table 1).

**Figure 1:**
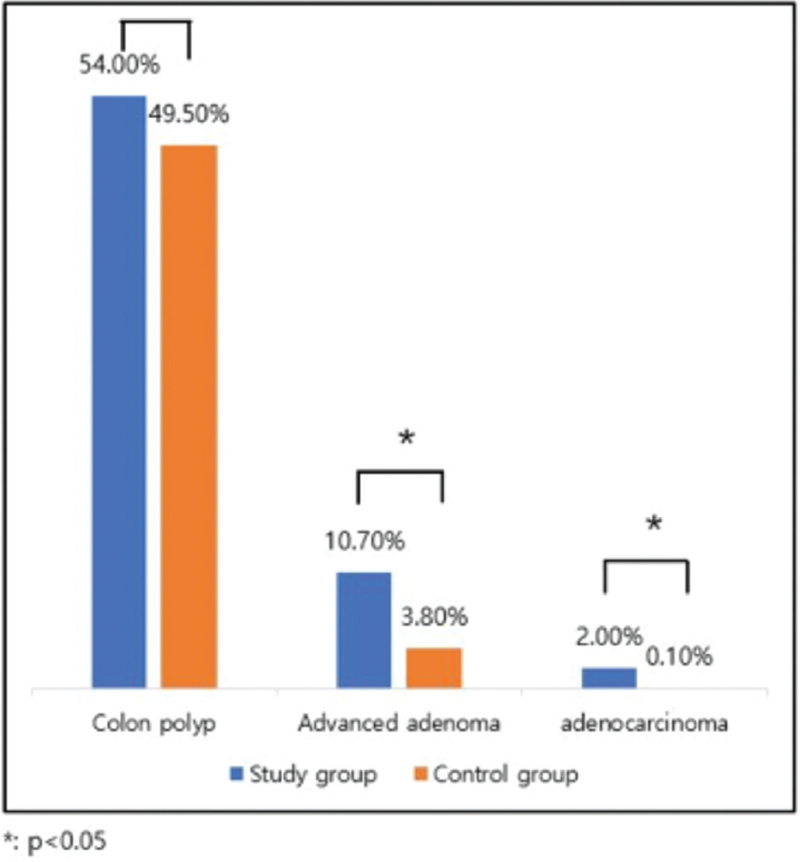
Prevalence of all colon polyps, advanced adenoma, and adenocarcinomas in the gastric neoplasm(study group) and control group. The prevalence of advanced adenoma and adenocarcinoma was significantly higher.

The prevalence of overall colorectal polyps and their distribution did not differ between the two groups (54.0% vs 49.5%, *P* = .079). However, the number and maximum size of colon polyps were significantly higher in the study group than in the control group. The prevalence of advanced colon adenoma was higher in the study group (10.7% vs 3.8%, *P* < .001). Pathological examination revealed tubular adenoma in 302 (42.9%), tubulovillous/villous adenoma in 19 (2.7%), serrated adenoma in 15 (2.1%), and adenocarcinomas in 14 (2.0%) patients in the study group, whereas examinations revealed 292 (36.6%) tubular adenomas, 10 (1.3%) tubulovillous/villous adenoma, 16 (2.0%) serrated adenoma, and 1 (0.1%) serrated adenoma in the control group. The prevalence of advanced adenoma and adenocarcinomas was significantly higher in the study group than in the control group (Fig. 1).

### 3.1 Risk factors for colon polyps and advanced colon adenoma

Risk factors for colon polyps and advanced colonic adenoma were evaluated using univariate and multivariate analyses. Univariate analysis showed that old age (58.14 ± 8.19 vs 59.19 ± 8.05, *P* = .012), high BMI (*P* = .015), low HDL-cholesterol level (57.50 ± 17.81 vs 55.28 ± 15.64, *P* = .046), high fasting glucose level (96.62 ± 25.22 vs 100.41 ± 27.16, *P* = .011), and presence of gastric neoplasm (*P* = .033) are related with colon polyps. Moreover, the group with advanced colon adenoma had significantly more patients in the gastric neoplasm group (*P* < .001) and had high fasting glucose levels (98.05 ± 25.16 vs 107.00 ± 38.88, *P* = .004) (Table 2). In the multivariate analysis, risk factors for colorectal polyps in both groups (study and control) were high glucose levels (odds ratio [OR], 1.007; 95% CI, 1.001–1.014; *P* = .034) and presence of gastric neoplasm (OR: 1.570, 95% CI: 1.000–2.464, *P* = .050). For advanced colon adenoma, the risk factor was the presence of gastric neoplasm (OR, 2.788; 95% CI, 1.718–4.524; *P* < .001) (Table 3).

**Table 2:** Univariate analysis of risk factors for colon polyps and for advanced colonic adenoma in the whole group (including control).

	All polyps			Advanced colon adenoma		
	Absence (n = 728)	Presence (n = 777)	*P* value	Absence (n = 1399)	Presence (n = 106)	*P* value
Age (yr, mean ± SD)	58.14 ± 8.19	59.19 ± 8.05	**.012**	58.60 ± 8.13	59.77 ± 8.11	.152
Male, Sex	503 (69.1%)	541 (69.6%)	.823	962 (68.8%)	82 (77.4%)	.066
BMI			**.015**			.143
< 23	256 (36.5%)	215 (29.5%)		447 (33.4%)	24 (26.4%)	
23 to < 25	176 (25.1%)	215 (29.5%)		369 (27.6%)	22 (24.2%)	
≥ 25	270 (38.5%)	298 (40.9%)		523 (39.1%)	45 (49.5%)	
Serum total cholesterol			.768			.094
< 200 mg/dL	402 (57.3%)	414 (57.4%)		402 (57.3%)	414 (57.4%)	
200 – 240mg/dL	218 (31.1%)	231 (32.0%)		218 (31.1%)	231 (32.0%)	
≥ 240 mg/dL	82 (11.7%)	76 (10.5%)		82 (11.7%)	76 (10.5%)	
Low density lipoprotein (mg/dL; mean ± SD)	124.27 ± 35.06	125.37 ± 34.91	.634	124.70 ± 35.15	127.31 ± 31.32	.637
High density lipoprotein (mg/dL; mean ± SD)	57.50 ± 17.81	55.28 ± 15.64	**.046**	56.56 ± 16.53	52.74 ± 21.38	.141
Glucose (mg/dL; mean ± SD)	96.62 ± 25.22	100.41 ± 27.16	**.011**	98.05 ± 25.16	107.00 ± 38.88	**.004**
CEA (ng/mL; mean ± SD)	2.29 ± 2.72	2.62 ± 5.84	.262	2.43 ± 4.73	2.76 ± 2.13	.537
CA 19–9 (U/mL; mean ± SD)	9.35 ± 11.94	12.13 ± 46.99	.301	10.32 ± 32.29	17.17 ± 59.97	.195
Gastric pathology			**.033**			**< .001**
Control	403 (55.4%)	395 (50.8%)		768 (55.2%)	30 (28.3%)	
Adenoma	90 (12.4%)	115 (14.8%)		181 (13.0%)	21 (19.8%)	
EGC	156 (21.4%)	201 (25.8%)		319 (22.8%)	38 (35.8%)	
AGC	79 (10.9%)	66 (8.5%)		131 (9.4%)	17 (16.0%)	

AGC = advanced gastric cancer, BMI = body mass index, CA 19–9 = carbohydrate antigen 19–9, CEA = carcinoemcryonic antigen, CI = confidence interval, EGC = early gastric cancer, SD = standard deviation.

**Table 3:** Multivariate logistic regression analysis risk factors for colon polyps and advanced adenoma in the whole group (including control).

	All polyps	*P* value	Advanced colon adenoma	*P* value
	Odds ratio (95% CI)		Odds ratio (95% CI)	
Age	0.990 (0.970–1.010)	.333	1.014 (0.987- 1.041)	.320
Sex		.561		.169
Male	Reference		Reference	
Female	1.101 (0.820–1.477)		0.687 (0.403 - 1.173)	
BMI		.977		
<25	Reference			
≥ 25	0.996 (0.754 -1.316)			
High density lipoprotein	1.006 (0.998–1.015)	.152		
Glucose	1.007 (1.001 - 1.014)	**.034**	1.005 (0.998 - 1.011)	.193
Gastric pathology		**.050**		**< .001**
Control	Reference		Reference	
Study group	1.570 (1.000 - 2.464)		2.788 (1.718 - 4.524)	

BMI = body mass index.

Based on the analysis of the study group, patients with colon polyps were significantly older (57.90 ± 10.28 vs 59.67 ± 9.56 years, *P* = .019). However, the risk factors for advanced colon adenoma have not been identified (Table 4).

**Table 4:** Univariate analysis of risk factors for colon polyps and for advanced colonic adenoma in the study group.

	All polyps			Advanced colon adenoma		
	Absence (n = 325)	Presence (n = 382)	*P* value	Absence (n = 631)	Presence (n = 76)	*P* value
Age (yr, mean ± SD)	57.90 ± 10.28	59.67 ± 9.56	.019	58.68 ± 10.05	60.34 ± 8.77	.167
Male, Sex	235 (72.1%)	259 (68.0%)	.236	436 (69.1%)	58 (76.3%)	.197
BMI			.913			.187
< 23	95 (31.7%)	110 (33.1%)		190 (33.3%)	15 (33.1%)	
23 to < 25	87 (29.0%)	96 (28.9%)		167 (29.2%)	16 (26.2%)	
≥ 25	118 (39.3%)	126 (38.0%)		214 (37.5%)	30 (49.2%)	
Serum total cholesterol			.201			.136
< 200 mg/dL	215 (71.7%)	245 (75.4%)		407 (72.4%)	53 (84.1%)	
200 – 240mg/dL	67 (22.3%)	70 (21.5%)		128 (22.8%)	9 (14.3%)	
≥ 240 mg/dL	18 (6.0%)	10 (3.1%)		27 (4.8%)	1 (1.6%)	
Low density lipoprotein (mg/dL; mean ± SD)	118.50 ± 40.10	113.48 ± 35.08	.473	115.79 ± 37.71	114.58 ± 34.60	.915
High density lipoprotein (mg/dL; mean ± SD)	57.14 ± 31.73	55.75 ± 19.42	.762	55.82 ± 24.54	61.83 ± 35.81	.446
Glucose (mg/dL; mean ± SD)	108.17 ± 29.87	108.41 ± 33.73	.934	107.63 ± 30.30	114.00 ± 44.58	.183
CEA (ng/mL; mean ± SD)	2.86 ± 3.97	3.20 ± 8.76	.577	3.08 ± 7.32	2.70 ± 1.98	.702
CA 19–9 (U/mL; mean ± SD)	9.29 ± 12.01	15.53 ± 73.36	.391	11.76 ± 52.88	20.22 ± 75.66	.400
Gastric pathology			.064			.869
Adenoma	90 (27.6%)	115 (30.1%)		181 (28.7%)	21 (27.6%)	
EGC	156 (48.1%)	201 (52.8%)		319 (50.6%)	38 (50%)	
AGC	79 (24.2%)	66 (17.0%)		131 (20.8%)	17 (22.4%)	

AGC = advanced gastric cancer, BMI = body mass index, CA 19–9 = carbohydrate antigen 19–9, CEA = carcinoemcryonic antigen, EGC = early gastric cancer, SD = standard deviation.

## 4 Discussion

Although advanced diagnostic modalities and techniques have enabled early detection of cancer, and the development of minimally invasive cancer treatments has enhanced the survival rate of cancer patients, their long-term survival increases the possibility of developing other primary malignancies. Studies in the East Asian population have shown that the stomach and colon are the most common sites of synchronous cancer.^[4,6,11]^ Although evidence confirming adenocarcinoma progression is limited, gastric adenoma gradually indicates malignant changes,^[12]^ and colon adenoma is a premalignant lesion that tends to be a colon cancer from the traditional adenoma–carcinoma sequence.

Recently, preoperative colonoscopy for patients with gastric adenocarcinoma has been recommended.^[13–15]^ Furthermore, a recent study showed that the overall survival of GC patients with colon adenoma or colon cancer was poorer than that of patients without surgically treated GC patients.^[16]^

The results of this study showed that the frequency of overall colon polyps was 54% in the gastric neoplasm group, including gastric adenoma, EGC, and AGC, and 49.5% in the sex-/age-matched control group. The results of this study differed from those of another previous study. In addition, several differences distinguish the present study from previous studies.

The first difference is that the number and size of colon polyps were higher in the gastric neoplasm group. Several studies have reported that there is no difference in the number and size of colon polyps between the two groups.^[17,18]^ In our study, the number of colon polyps (1.20 ± 1.71 vs 0.99 ± 1.54, *P* = .015) and the maximal size (3.53 ± 6.14 vs 2.08 ± 2.88, *P* < .001) were significantly larger in the gastric neoplasm group. The large number and size of colon polyps were associated with advanced adenoma, especially if the number of colon polyps was 3 or more or the size of the colon polyps was 10 mm or more, although the colonoscopy interval was reduced.^[19,20]^ In this regard, the fact that the number and size of colon polyps in the gastric neoplasm group is larger is significant, as it suggests the possibility of developing a second primary cancer in patients treated with gastric neoplasm.

The second difference was the prevalence of colon adenocarcinoma between the two groups. Similar to previous studies, the prevalence of high-risk advanced colon adenoma was 10.7% in the gastric neoplasm group and 3.8% in the control group, respectively, similar to previous studies (*P* < .001). However, colon adenocarcinoma was found in 14 patients (2.0%) in the gastric neoplasm group and in one patient (0.1%) in the control group, which is different from previous reports where there was no statistical difference (*P* = .015).

In this study, significant risk factors for overall colon polyps and advanced colon adenomas were determined using a multivariate logistic regression model. For all colon polyps, the risk factors included glucose level and the presence of gastric neoplasm. The only risk factor for advanced colon adenoma was the presence of gastric neoplasms. In the subgroup analysis of patients with gastric neoplasms, only age was significantly associated with colon polyps. The mechanism underlying the close association between gastric and colorectal cancers is unknown. Several factors support the association between gastric and colorectal neoplasms. Some have hypothesized that these phenomena are caused by *Helicobacter pylori* infections,^[13,15]^ and several studies testing this hypothesis have been conducted. *Helicobacter pylori* infection is associated with increased secretion of gastrin, which could be a trophic factor in the colonic mucosa. Some studies have suggested that genetic alteration and microsatellite instability play a role.^[8,9,18,21]^ This suggests that the association between GC and colon cancer is a genetic correlation. hMSH1 and hMSH2 genes play important roles in repairing base-pair mismatches found during DNA replication, and changes in these genes are important in the incidence of hereditary nonpolyposis colon cancer.

Adenomatous polyps have been widely accepted as precursors of colon cancer, and their removal is important for decreasing the incidence and mortality of colon cancer.^[22,23]^ In patients with advanced colorectal adenoma on the first diagnostic colonoscopy, the prevalence of metachronous colorectal neoplasm was approximately 10 times higher than the general population.^[20]^ In addition, the presence of advanced colon adenoma has increased the risk of synchronous advanced colon polyps in other parts of the colon in patients with advanced colon adenoma.^[24]^

Some reports have demonstrated a relationship between patients with GC who underwent gastrectomy and colonoscopy.^[13,25]^ Preoperative colonoscopy is recommended for patients with GC because of the high prevalence of colorectal neoplasms, including colon cancer, compared to the normal population. Performing colonoscopy after gastrectomy may be difficult due to adhesions, poor preparation, and anatomical changes.

The primary limitation of the current study is its retrospective nature. Many relative factors, such as current smoking, current moderate to heavy alcohol use, physical activity, supplements, and chemopreventive agents, could not be assessed.^[26]^ However, other important variables, such as BMI, fasting glucose level, and lipid profiles (serum total cholesterol, HDL, and LDL) were included, and a relatively large number of patients were analyzed compared to previous retrospective studies. Second, it is possible that only individuals with health consciousness and financial independence were enrolled in the healthcare center as a control group. Of a total of 3067 gastric adenoma or gastric cancer patients, 2360 patients did not undergo colonoscopy, and only 707 patients underwent colonoscopy. 707 patients who underwent colonoscopy are more likely to have another risk factor for developing colon neoplasms compared to the remaining 2360 patients. This suggests that this could be a selection bias.

In conclusion, this study demonstrated that patients with gastric neoplasms had a significantly higher risk of colon adenomas. Advanced colon adenoma is associated with progression from benign adenomas through malignant altered adenomas to advanced colon cancer. Therefore, patients with gastric neoplasm can be considered a high-risk group for colorectal cancer, and colonoscopy is recommended at the time of diagnosis and in a short period of time.

## Author contributions

**Conceptualization:** Jin Seok Jang.

**Data curation:** Min-Chan Kim, Myeongseok Koh.

**Formal analysis:** Myeongseok Koh.

**Methodology:** Min-Chan Kim.

**Supervision:** Jin Seok Jang.

**Writing – original draft:** Myeongseok Koh.

**Writing – review & editing:** Myeongseok Koh.

## Supplementary Material

Supplemental Digital Content
