# Supplementary material for: Difference in the prevalence of advanced colon adenoma between patients with gastric neoplasm and healthy people: A STROBE-compliant study
Source: Medicine (Baltimore). 2022 May 27;101(21):e29308. doi: 10.1097/MD.0000000000029308 (PMC9276267; doi:10.1097/MD.0000000000029308)
Supplement: Supplemental Digital Content [file medi-101-e29308-s001.docx]

**Supplementary Table 1. Baseline Characteristics in the Study Group.**

|  | AGC (202) | EGC (357) | Adenoma (148) | P value |
| --- | --- | --- | --- | --- |
| Age (year, mean ± SD) | 60.57 ± 9.16 | 59.05 ± 9.93 | 56.04 ± 10.45 | **<0.001** |
| Male, Sex, n (%) | 134 (66.3%) | 252 (71.2%) | 105 (73.9%) | 0.278 |
| BMI (kg/m2; mean±SD) | 24.43 ± 3.12 | 23.92 ± 3.09 | 23.36 ± 3.30 | **0.014** |
| Total cholesterol (mg/dL; mean±SD) | 186.92 ± 150.49 | 178.36 ± 150.49 | 180.92 ± 38.64 | 0.578 |
| Low density lipoprotein (mg/dL; mean±SD) | 122.34 ± 31.93 | 116.49 ± 37.36 | 77.70 ± 42.49 | **0.002** |
| High density lipoprotein (mg/dL; mean±SD) | 59.16 ± 29.48 | 56.23 ± 22.94 | 45.44 ± 18.45 | 0.172 |
| Glucose (mg/dL; mean±SD) | 109.56 ± 39.86 | 108.35 ± 29.68 | 107.00 ± 25.89 | 0.838 |
| CEA (ng/mL; mean±SD) | 3.65 ± 11.37 | 2.81 ± 3.82 | 2.58 ± 3.10 | 0.369 |
| Colon polyp, (number, mean ± SD) | 1.25 ± 1.71 | 1.25 ± 1.78 | 1.01±1.54 | 0.343 |
| Colon polyp size (mm, mean ± SD) | 3.50 ± 5.43 | 3.70 ± 6.76 | 3.22 ± 5.57 | 0.729 |
| Advanced adenoma, n (%) |  |  |  | 0.869 |
| YES | 21 (10.4%) | 38 (10.6%) | 17(11.5%) |  |
| NO | 181 (89.6%) | 319(89.4%) | 131 (88.5%) |  |

SD, standard deviation; BMI, body mass index; CEA, carcinoembryonic antigen.
